# Enhanced Electrochemical Performance of Carbon-Composited Co_3_O_4_ Microspheres as Anode Materials for Lithium-Ion Batteries

**DOI:** 10.3390/ma17235702

**Published:** 2024-11-21

**Authors:** Achmad Yanuar Maulana, Jongsik Kim

**Affiliations:** 1 Department of Chemistry, Dong-A University, Busan 49315, Republic of Korea; 1776107@donga.ac.kr; 2 Department of Chemical Engineering (BK21 FOUR Graduate Program), Dong-A University, Busan 49315, Republic of Korea

**Keywords:** Li-ion batteries, cobalt oxide, anode, carbon-composited microspheres, abietic acid

## Abstract

Cobalt (II, III) oxide (Co_3_O_4_) has recently gained attention as an alternative anode material to commercial graphite in lithium-ion batteries (LIBs) due to its superior safety and large theoretical capacity of about 890 mAh g^−1^. However, its practical application is limited by poor electrical conductivity and rapid capacity degradation because of significant volume increases and structural strain during repeated lithiation/delithiation cycles. To address these issues, this work presents a novel approach to synthesizing carbon-composited Co_3_O_4_ microspheres (Co_3_O_4_@C), using abietic acid (AA) as a carbon source to increase conductivity and structural stability. The resulting Co_3_O_4_@C anodes show an impressive discharge capacity of 1557.4 mAh g^−1^ after 200 cycling processes at a current density of 0.1 C, representing a significant improvement over bare Co_3_O_4_. This study demonstrates the potential of carbon-compositing as a strategy to mitigate the limitations of Co_3_O_4_ and extend its cyclability, making it a viable candidate for next-generation LIB anodes.

## 1. Introduction

The growing demand for high-performance energy storage systems (EESs), particularly for electric vehicles and portable electronics, has spurred extensive research into alternative materials for lithium-ion batteries (LIBs) that offer greater capacity, longer lifespans, and improved safety compared to conventional LIBs [1]. Graphite, the most widely used anode material, has a low theoretical capacity of about 372 mAh g^−1^ and a low rate capability, which limits its potential in high-energy applications [2]. To address these shortcomings, numerous studies have explored some replacement anode materials, such as Li_3_VO_4_ and TiO_2_ as intercalation-type anodes, Si and Bi as alloying-type anodes, and ZnO, Fe_3_O_4_, and MoO_2_ as conversion-type anodes [3,4,5,6,7,8,9]. However, while intercalation-type materials generally offer good cycling stability, the intercalation anodes are constrained by their lower theoretical capacities (less than 400 mAh g^−1^) [10]. Alloying-type materials, though capable of high theoretical capacities (up to 4200 mAh g^−1^), suffer from significant volumetric expansion during cycles, leading to poor cycling stability [11]. In contrast, transition metal oxides (TMOs) offer a promising alternative, offering higher theoretical capacities (ranging from 375 to 1058 mAh g⁻^1^) compared to intercalation-type anodes and more moderate volume increases than alloying-type materials [12].

Among TMOs, cobalt oxide (Co_3_O_4_) stands out because of its simple synthesis processes, high theoretical capacity (890 mAh g^−1^) through Co_3_O_4_ + 8Li^+^ + 8e^−^ ↔ 3Co + 4Li_2_O, and favorable operating voltage (~1.0 V relative to Li/Li^+^), which can reduce the risk of lithium dendrite formation and thus enhances safety [13,14]. In practical applications, Co_3_O_4_ faces limitations as an anode material due to rapid capacity fading and low rate performance, which are primarily attributed to its low electrical and ionic conductivities with substantial volume increases during repeated charge/discharge cycles [13,14]. To overcome these challenges, Co_3_O_4_ has been integrated with conductive carbon like graphene, hard carbon, and carbon nanotubes (CNTs) to enhance electrical conductivity along with maintaining structural stability during cycling [14,15,16]. For instance, Wang et al. synthesized Co_3_O_4_-graphene oxide (GO) composites and demonstrated a large capacity of 851.3 mAh g⁻^1^ after 250 discharges at 0.1 A g^−1^, owing to the synergistic effect of GO in improving electron conductivity and buffering volume changes during cycling [17]. Similarly, Wu et al. developed hollow Co_3_O_4_ nanocrystals in situ anchored on holey graphene, resulting in good cyclability, with a reversible discharge capacity of 840 mAh g^−1^ after 250 cycles at 0.2 A g^−1^ [18]. Despite these advances, achieving optimal electrochemical performance remains a challenge due to the difficulty of simultaneously nanostructuring Co_3_O_4_ and effectively integrating it with carbon matrices at the molecular level from the first synthesis step.

**Figure 1:**
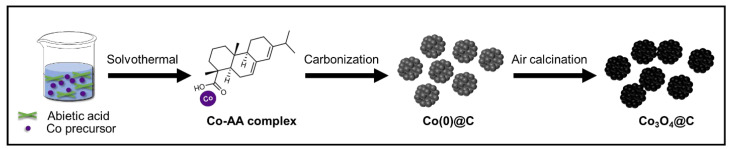
Preparation of the Co_3_O_4_@C microspheres.

In this study, we report the synthesis of carbon-composited Co_3_O_4_ microspheres (Co_3_O_4_@C) using abietic acid as a carbon source as shown in Figure 1. Abietic acid (C_20_H_30_O_2_), a natural resin derived from pine trees, not only is environmentally friendly and cost-effective but also has a hydrogenated phenanthrene structure that provides similar characteristics to petroleum-based aromatic compounds [19]. These characteristics make abietic acid an excellent precursor for creating conductive graphitic carbon matrices. In addition, the carboxylic groups in AA can attract cationic ions like Co^2+^ and Co^3+^ to the formation of the Co-AA complex [20]. By leveraging its unique microsphere morphology, the synthesized Co_3_O_4_@C anodes exhibited significantly enhanced electrochemical performances, with discharge capacity of 1557.4 mAh g^−1^ even after 200 cycles at 0.1 C. This improvement underscores the effectiveness of graphitic carbon composites in enhancing the structural stability and electronic conductivity of metal oxide anodes, demonstrating the potential of graphitic carbon-composited Co_3_O_4_ microspheres as a promising alternative for future energy storage technologies.

## 2. Materials and Methods

### 2.1. Sample Preparation

The Co_3_O_4_@C was synthesized by mixing 0.291 g (1 mmol) of Co(NO_3_)_2_∙6H_2_O (98%, Sigma Aldrich, St. Louis, MO, USA) and 0.3025 g (1 mmol) of AA (Alfa Aesar, Haverhill, MA, USA) in 30 mL of ethanol (1:1 m/m), followed by stirring for about 1 h and placed in a 50 mL Teflon-lined stainless-steel autoclave for solvothermal reaction at 180 °C for 12 h. The obtained Co-AA complex was filtered, washed with ethanol, and dried at 80 °C inside a dry oven overnight. Then, the as-prepared sample was carbonized under flowing Ar gas (0.5 L min^−1^) at 800 °C for 3 h with a heating rate of 5 °C min^−1^. Finally, the Co_3_O_4_@C microspheres were obtained by air calcination at 350 °C for 12 h. The variable sample of Co_3_O_4_@C*2 was prepared by increasing the AA amount to 0.605 g (2 mmol) with a similar route as Co_3_O_4_@C fabrication. For comparison, bare Co_3_O_4_ was prepared by calcining Co(NO_3_)_2_·6H_2_O at 700 °C for 3 h under air conditions.

### 2.2. Characterization

#### 2.2.1. Material Characterization

The particle size and morphology of the samples were examined using transmission electron microscopy (TEM, JEOL JEM-2011, Tokyo, Japan) and scanning electron microscopy (SEM, JEOL JSM-6700F). Fourier transform infrared (FT-IR) spectra were recorded using a JASCO FT-IR-4600 spectrometer (Tokyo, Japan), while Raman spectra were collected using a WITec alpha 300R (Kroppach, Germany) with a 532 nm He-Ne laser source. X-ray diffraction (XRD) analysis was measured using a Rigaku Miniflex 600 X-ray diffractometer (Cu Kα radiation (λ) = 1.5418 Å, Tokyo, Japan). Surface composition was observed through X-ray photoelectron spectroscopy (XPS) with a Theta Probe AR-XPS system by using the Al Kα X-ray source (hv = 1486.6 eV). The binding energies were calibrated with C1s core level peak at 284.6 eV. The XPS spectra were fitted using the XPSPEAK41 software (version 4.1) with the background type of “Shirley” by selecting an “s” peak shape for the analysis. The carbon content of the samples was analyzed by thermogravimetric analysis (TGA) with a SETARAM SETSYS Evolution system, conducted in air at a temperature of 20 to 800 °C, with a heating increment of 5 °C per minute. The Brunauer–Emmett–Teller (BET) analysis was performed on a Micromeritics ASAP 2010 gas sorption analyzer (Norcross, GA, USA) under a nitrogen (N_2_) atmosphere.

#### 2.2.2. Electrochemical Measurements

Electrochemical performances were tested using 2032-type coin cells. Working electrodes were fabricated by blending the active material with acetylene black (MTI) and polyvinylidene fluoride (PVDF, Sigma-Aldrich) in a 70:20:10 weight ratio, employing N-methylpyrrolidone (NMP, Sigma-Aldrich) as the solvent. The prepared slurry was uniformly applied onto copper foil (30 µm thick) and dried in a vacuum oven at 80 °C for 30 min. Once dried, electrodes were punched into circular disks (14 mm in diameter) and then vacuum-dried at 80 °C for 2 h. The electrode loading was controlled to be about 1.8 mg cm^−2^. A pure lithium metal counter electrode was utilized, and a polypropylene membrane (Celgard 2400, Charlotte, NC, USA) served as the separator. The electrolyte consisted of a 1.0 M LiPF₆ solution in ethylene carbonate and dimethyl carbonate (EC:DMC = 1:1 volume ratio, Panaxetec, Busan, Republic of Korea). The coin cell preparations took place in an argon-filled glove box. The electrochemical properties were evaluated using a WonATech WBCS3000 system (Seoul, Republic of Korea) within a voltage range of 0.01–3.0 V at room temperature. Electrochemical impedance spectroscopy (EIS) was performed over a frequency range of 10^−2^ to 10^5^ Hz using a ZIVE SP2 impedance analyzer (WonATech). All electrochemical cell tests were conducted at a temperature of 30 °C.

## 3. Results and Discussion

### 3.1. Structural and Morphological Characterization

**Figure 2:**
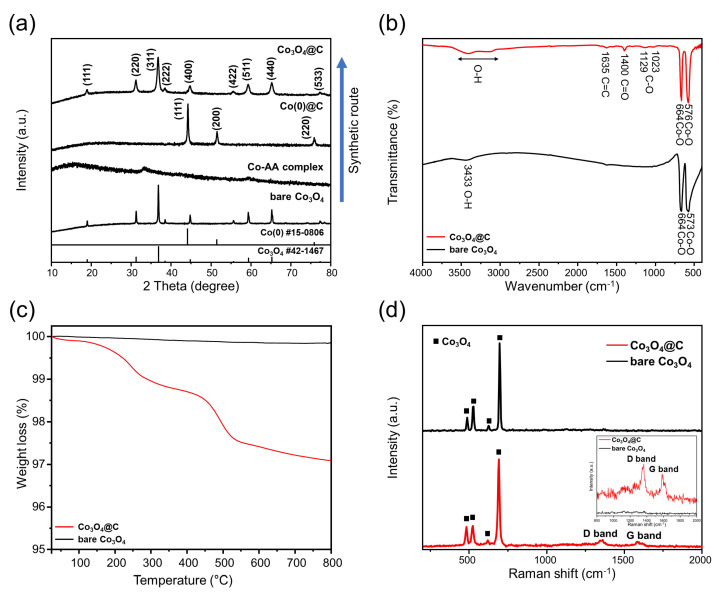
(**a**) XRD of bare Co_3_O_4_, Co_3_O_4_@C, and intermediate products of Co_3_O_4_@C synthetic route; (**b**) FT-IR, (**c**) TGA, and (**d**) Raman spectra of bare Co_3_O_4_ and Co_3_O_4_@C.

Figure 2a and Figure S1 present the XRD spectra of bare Co_3_O_4_ and the intermediate products from the fabrication of Co_3_O_4_@C and Co_3_O_4_@C*2. The reflections from the bare Co_3_O_4_ sample and the Co_3_O_4_@C and Co_3_O_4_@C*2 samples can be indexed to a cubic spinel structure with the Fd3m space group (JCPDS card No. 42–1467) [21]. During the carbonization of the Co-AA complex at 800 °C, Co ions were reduced to Co(0). The broader XRD peaks of the Co_3_O_4_@C and Co_3_O_4_@C*2 samples compared to the bare Co_3_O_4_ suggest a smaller particle size. This observation was further analyzed by particle size calculations from the Debye–Scherrer equation, with unit sizes for the bare Co_3_O_4_ sample and the Co_3_O_4_@C and Co_3_O_4_@C*2 samples estimated at 50.6, 35.6, and 32.8 nm, respectively. The smaller particle size in the Co_3_O_4_@C and Co_3_O_4_@C*2 samples indicates that the carbon layers play a crucial role in preventing particle growth during the synthesis process.

The FT-IR graph of the bare Co_3_O_4_ sample and the Co_3_O_4_@C sample is presented in Figure 2b. Both samples exhibit peaks at 3400 cm^−1^, corresponding to the vibrational modes of adsorbed H_2_O [13]. Vibrational modes at 664 and 576 cm^−1^ were assigned to Co–O bonds [13]. In contrast, the Co_3_O_4_@C sample exhibited additional peaks at 1635, 1400, and 1129 and 1023 cm^−1^, which correspond to the vibrations of C=C, C=O, and C–O bonds, respectively [22]. The presence of the C=C bond suggests the formation of sp^2^ carbon structures, which are known to improve the electrical conductivity of the sample [23].

Figure 2c illustrates the thermogravimetric analysis (TGA) curves for the bare Co_3_O_4_ sample and the Co_3_O_4_@C sample to observe the carbon content of the Co_3_O_4_@C sample. The bare Co_3_O_4_ exhibited a small weight loss (~0.14 wt%) due to the evaporation of surface-adsorbed water. In comparison, the Co_3_O_4_@C sample exhibited a weight loss of about 1.23 wt% between 20 °C and 350 °C, attributed to the adsorbed water from ambient humidity and possible minor decomposition of surface hydroxyls. The subsequent weight loss of about 1.68 wt% was obtained between 350 °C and 800 °C, indicating the decomposition of carbon [19]. Based on these results, the total carbon content in the Co_3_O_4_@C sample is estimated to be around 1.68 wt%. Further investigation into the carbon structure of the samples was conducted using Raman spectroscopy, as shown in Figure 2d. The bare Co_3_O_4_ sample did not exhibit any distinct signals between 1350 cm^−1^ and 1580 cm^−1^, confirming the absence of carbon structures. However, the signals that the Co_3_O_4_@C sample displayed were observed near 1350 and 1580 cm^−1^, corresponding to the disordered (D) carbon structure of sp^3^ and the graphitic (G) carbon structure of sp^2^, respectively [24]. The D-to-G-band ratio (I_D_/I_G_) in the Co_3_O_4_@C sample indicates a slightly higher D-band intensity, which suggests the presence of defects or disorder in the sp^2^ structure. These defects can facilitate the efficient transport of positive charge carriers and electrolytes, which is advantageous in anode material applications. Additionally, both the bare Co_3_O_4_ sample and the Co_3_O_4_@C sample showed peaks at 480, 518, 621, and 690 cm^−1^, corresponding to the E_g_, two F_2g_, and A_1g_ vibrational modes of Co_3_O_4_ [25].

**Figure 3:**
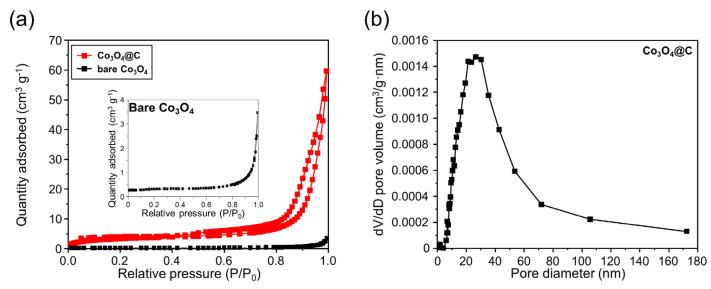
(**a**) N_2_ adsorption/desorption isotherm and (**b**) pore size distributions of bare Co_3_O_4_ and Co_3_O_4_@C.

The surface area and pore properties of bare Co_3_O_4_ and Co_3_O_4_@C were evaluated using BET analysis, as presented in Figure 3. The bare Co_3_O_4_ sample exhibits an IUPAC type II isotherm with the absence of a hysteresis loop and is typically associated with nonporous materials, as seen in Figure 3a [24,26]. On the other hand, Co_3_O_4_@C represents the IUPAC type II with an H3-type hysteresis loop, demonstrating the macroporous structure with wedged pore shapes [24,26]. The surface area of Co_3_O_4_@C was determined to be ~11.89 m^2^ g^−1^, which is significantly ten times higher than the bare Co_3_O_4_ sample (~1.18 m^2^ g^−1^). Moreover, the pore size of Co_3_O_4_@C ranges from 5 to 75 nm, with the most common pore size being around 35 nm, and an average pore volume of about 0.092 cm^3^ g^−1^, as illustrated in Figure 3b. This macroporosity in the Co_3_O_4_@C microspheres sample is crucial in enhancing electrolyte penetration and improving the efficiency of Li-ion insertion and extraction during cycling, which ultimately benefits the electrochemical performance of the material.

**Figure 4:**
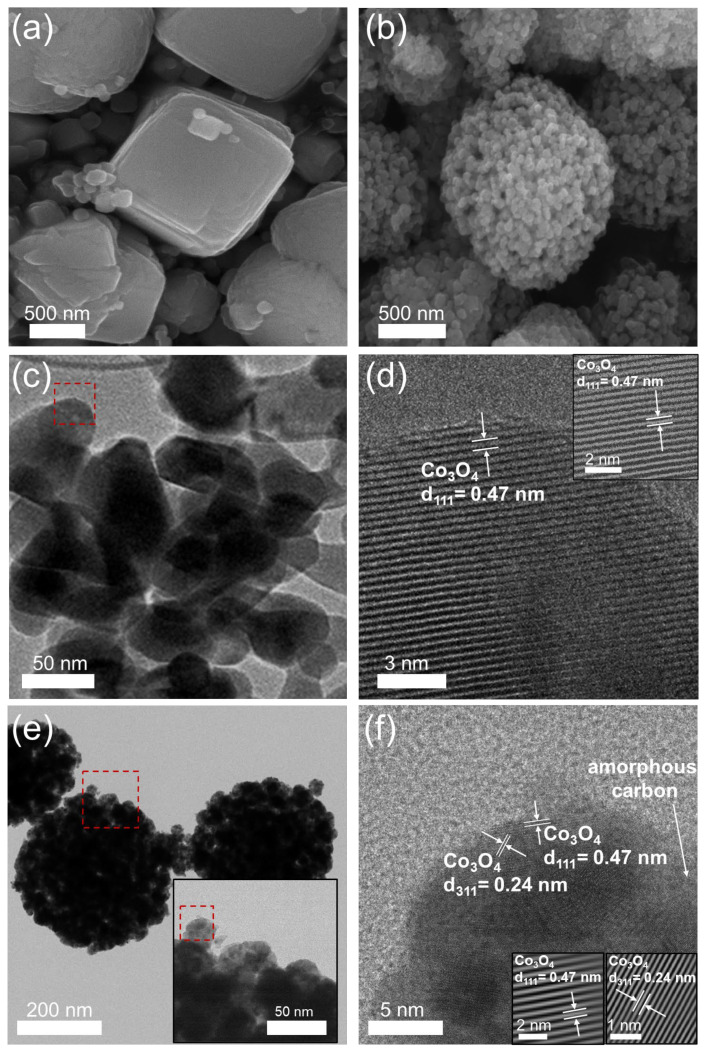
SEM images of (**a**) bare Co_3_O_4_ and (**b**) Co_3_O_4_@C; (**c**) TEM and (**d**) HRTEM images of bare Co_3_O_4_; (**e**) TEM and (**f**) HRTEM images of Co_3_O_4_@C. (Inset images: FFT patterns).

The morphologies of bare Co_3_O_4_ and Co_3_O_4_@C, obtained after air calcination, were examined using SEM and TEM, as presented in Figure 4. The SEM image of bare Co_3_O_4_ in Figure 4a reveals cubical slab-shaped particles with lengths ranging from 1 to 1.3 µm and widths between 0.9 and 1.2 µm. Conversely, Figure 4b shows that Co_3_O_4_@C has a significantly smaller particle size, with diameters of about 45 to 53 nm, forming granular shapes that clustered to create a larger microsphere with diameters of about 1 to 1.2 µm. The TEM images of bare Co_3_O_4_ in Figure 4c,d present ellipsoidal particles with an average length of about 75 nm and 52 nm in width. High-resolution TEM (HRTEM) of bare Co_3_O_4_ displays lattice fringes with a d-spacing of 0.47 nm, associated with the (111) planes of the cubic spinel Co_3_O_4_ crystal structure [27]. In contrast, the TEM images of the Co_3_O_4_@C sample in Figure 4e,f reveal that the morphology has transformed into nanosized spheres with a diameter size of about 23 to 31 nm compared to bare Co_3_O_4_. The HRTEM analysis of Co_3_O_4_@C in Figure 4f shows distinct lattice fringes with a d-spacing of 0.24 nm from the (311) plane and 0.42 nm from the (111) plane of the cubic spinel Co_3_O_4_ crystal structure [28]. Additionally, the presence of amorphous carbon in the Co_3_O_4_@C can enhance the conductivity of Co_3_O_4_ and provide additional sites for Li-ion storage. The lattice fringes were further confirmed through Fourier-filtered transform (FFT) images of the magnified HRTEM, as shown in the insets of Figure 4d,f.

**Figure 5:**
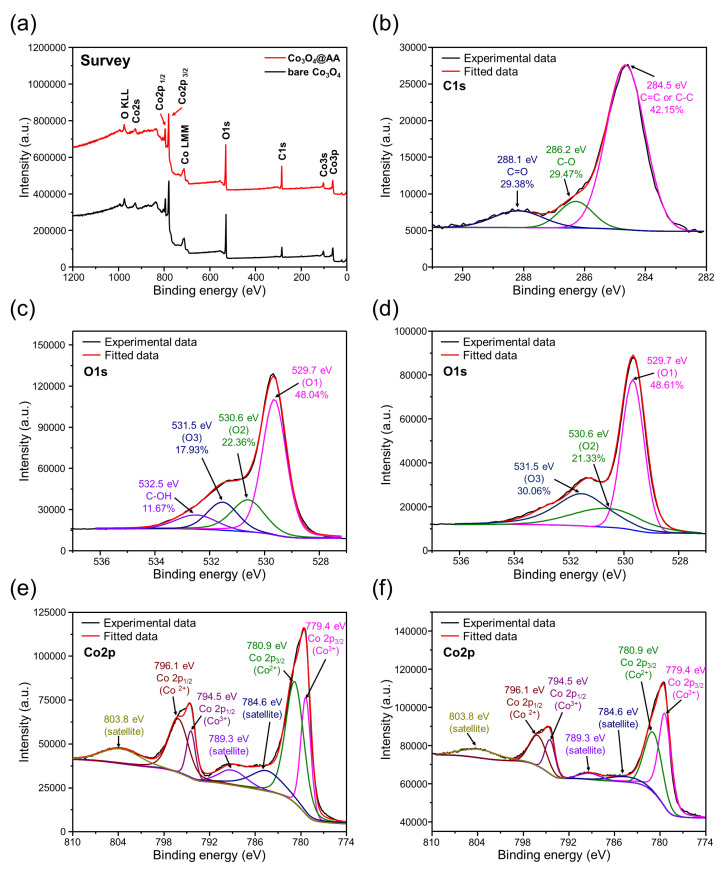
(**a**) XPS spectra of bare Co_3_O_4_ and Co_3_O_4_@C. XPS region of (**b**) C1s for Co_3_O_4_@C; O1s regions for (**c**) Co_3_O_4_@C and (**d**) bare Co_3_O_4;_ Co2p regions for (**e**) Co_3_O_4_@C and (**f**) bare Co_3_O_4_.

The chemical compositions on the surfaces of the bare Co_3_O_4_ sample and the Co_3_O_4_@C sample were analyzed using XPS, as shown in Figure 5. As shown in Figure 5a, the overall XPS profiles of both samples display peaks corresponding to Co_3_p, Co_3_s, Co_2_p_3_/_2_, Co_2_p_1_/_2_, and O_1_s, confirming that Co_3_O_4_ is the primary component. However, the Co_3_O_4_@C sample also exhibits a pronounced signal in the C_1_s region, reflecting the presence of carbon complex. The C_1_s spectrum, shown in Figure 5b, can be deconvoluted into three distinct peaks at binding energies of 284.5, 286.2, and 288.1 eV, which are associated with C=C/C–C, C–O, and C=O bonds, respectively [29]. The predominant C=C/C–C bonds can positively influence the electrochemical performances of the electrode in LIB application [30].

The O1s region of both the Co_3_O_4_@C and bare Co_3_O_4_ samples, depicted in Figure 5c,d, reveals three similar peaks at 529.7, 530.6, and 531.5 eV, corresponding to O_1_, O_2_, and O_3_, respectively [13,31]. These peaks are attributed to Co–O bonds (O_1_), Co–OH bonds (O_2_), and surface-adsorbed moisture (O3). The existence of an additional peak at 532.5 eV is observed for the Co_3_O_4_@C sample, which is attributed to C–O(H) bonds of the carbon matrix [32]. This additional peak indicates the presence of carbon that is functionalized with hydroxyl groups (–OH), likely due to the carbon composites formed during synthesis. The appearance of the C–OH peak in the Co_3_O_4_@C sample confirms the successful integration of carbon into the composite material. Furthermore, Figure 5e,f show the Co_2_p region for both Co_3_O_4_@C and bare Co_3_O_4_, with characteristic doublets for Co_2_p_3_/_2_ and Co_2_p_1_/_2_ observed at around 778–781 eV and 793–796 eV, respectively [33]. The peaks at 779.4 eV and 794.5 eV are attributed to Co^3+^ species, while the peaks at 780.9 eV and 796.1 eV correspond to Co^2+^ species [33]. This indicates that cobalt ions in both samples exist in two oxidation states of +2 and +3. Additionally, satellite peaks are observed at 784.6 eV, 789.3 eV, and 803.8 eV, further confirming the electronic structure of the cobalt ions [13].

### 3.2. Electrochemical Performance

**Figure 6:**
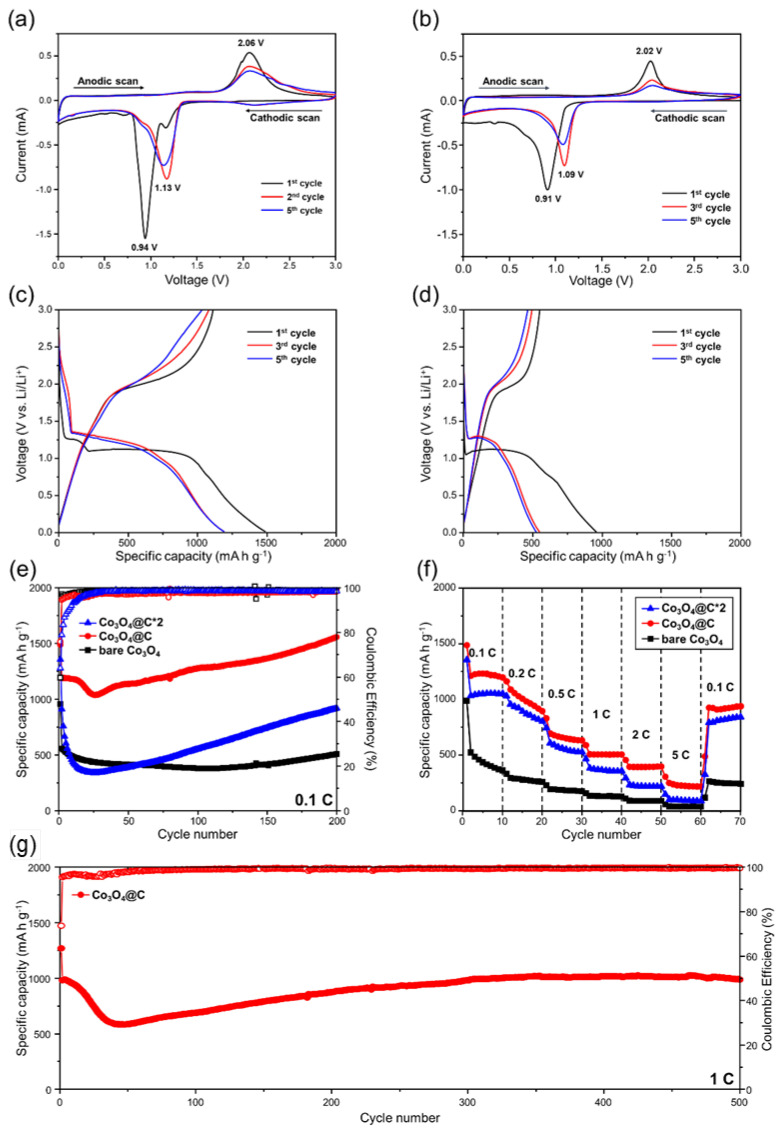
CV curve at a scan rate of 0.1 mV s^−1^ from (**a**) Co_3_O_4_@C and (**b**) bare Co_3_O_4_ in the voltage range of 0.1–3.0 V; galvanostatic discharge/charge profiles of (**c**) Co_3_O_4_@C and (**d**) bare Co_3_O_4_ at 0.1 C using a voltage range of 0.01–3.0 V; (**e**) cycling performance at 0.1 C and (**f**) rate capability at various current densities for bare Co_3_O_4_, Co_3_O_4_@C, and Co_3_O_4_@C*2; (**g**) high rate experiment of Co_3_O_4_@C at 1 C.

Figure 6a,b show the fifth cyclic voltammetry (CV) profiles of the Co_3_O_4_@C and bare Co_3_O_4_ samples at a scan rate of 0.1 mV s^−1^. During the initial cathodic scan, both samples exhibit peaks at around 0.9 V, indicating the reduction of Co_3_O_4_ to Co(0) and Li_2_O [34]. An additional weak peak at 1.16 V in the Co_3_O_4_@C sample corresponds to the generation of the solid electrolyte interphase (SEI) layer, which is more pronounced in the carbon composite material compared to the bare Co_3_O_4_ [34]. In the initial anodic scan, both samples display a peak at around 2.03 V, representing the oxidation of Co(0) and Li_2_O back to Co_3_O_4_ [35]. On the second cathodic scan of the Co_3_O_4_@C sample, the peaks broaden and shift to a higher potential which might be because of the activation of the electrode during the initial cycle [13,36]. Interestingly, bare Co_3_O_4_ also shows a shifted peak with a slightly lower potential (1.09 V) compared to Co_3_O_4_@C (1.13 V), indicating that the redox reaction occurs more easily in the Co_3_O_4_@C sample. Moreover, the larger CV area for Co_3_O_4_@C suggests a higher discharge capacity compared to bare Co_3_O_4_.

Figure 6c presents the initial two galvanostatic discharge/charge curves for Co_3_O_4_@C at 0.1 C within a voltage range of 0.01–3.0 V. In the initial discharge process, a voltage plateau of ~1.2 V occurs, suggesting the formation of SEI layers [34]. Another plateau at ~1.0 V is associated with the conversion reaction of Co_3_O_4_ to Co(0) and Li_2_O [34]. In subsequent cycles, the plateau shifts slightly to a higher voltage. The bare Co_3_O_4_ sample in Figure 6d also shows similar results with the missing plateau of SEI formation compared to Co_3_O_4_@C. These findings align well with the CV results above. During the initial discharge, Co_3_O_4_@C delivers a capacity of about 1488.6 mAh g^−1^, with an initial capacity loss of around 20.09%, mainly due to irreversible SEI formation. These values represent a significant improvement over bare Co_3_O_4_, which delivers a capacity of about 956.9 mAh g^−1^ with a higher initial capacity loss of 41.50%. These results also outperform recent reports, where initial capacity losses range from 30% to 47% [37,38,39,40,41].

The cycling performance of bare Co_3_O_4_, Co_3_O_4_@C, and Co_3_O_4_@C*2 was examined at a current rate of 0.1 C and a voltage range of 0.01-3.0 V, as presented in Figure 6e. It can be seen that the discharge capacities of Co_3_O_4_@C and Co_3_O_4_@C*2 gradually increase after an initial decline up to the 25th cycle, likely due to electrode activation. Additionally, the bare Co_3_O_4_ sample also shows a slight capacity increase after around 155 cycles compared to Co_3_O_4_@C. This trend of increasing capacity in transition metal oxide (TMO)-based anodes is commonly attributed to the establishment of a gel-like polymeric film [39,41,42]. After 200 cycles, the reversible discharge capacities of bare Co_3_O_4_, Co_3_O_4_@C, and Co_3_O_4_@C*2 are 499.4, 1557.4, and 919.9 mAh g^−1^, respectively. The higher capacities of the Co_3_O_4_@C and Co_3_O_4_@C*2 samples exceeding the theoretical value of 890 mAh g^−1^ are likely due to additional Li-ion storage sites at the grain boundaries of Li_2_O and Co, generated throughout the discharge process [43]. The unique Co₃O₄@C microspheres may also contribute to the extra active sites for Li-ion storage. The lower reversible discharge capacity of Co_3_O_4_@C*2 compared to Co_3_O_4_@C suggests that the higher carbon content in Co_3_O_4_@C*2 results in a lower concentration of active Co_3_O_4_, which contributes more to the capacity. The Coulombic efficiencies (CEs) of the entire samples presented more than 91% after the initial cycle, indicating stable performance. The cycling performance of Co_3_O_4_@C is competitive with previous studies, as summarized in Table S1 [13,17,18,41,43,44,45,46].

Figure 6d exhibits the rate capability of the bare Co_3_O_4_ sample and the Co_3_O_4_@C and Co_3_O_4_@C*2 samples. It can be seen that the Co_3_O_4_@C sample delivers reversible discharge capacities of 1015.3, 891.7, 497.2, 310.4, 198.2, and 52.8 mAh g^−1^ at current rates of 0.1, 0.2, 0.5, 1, 2, and 5 C, respectively. These values exceed those observed for bare Co_3_O_4_ and Co_3_O_4_@C*2, with the discharge capacities corresponding to the second cycle at each rate. Upon reducing the current density back to 0.1 C, Co_3_O_4_@C exhibited a large recovery rate of 79.7%, compared to 68.1% for bare Co₃O₄ and 77% for Co_3_O_4_@C*2. This demonstrates the robust structure of the Co_3_O_4_@C electrode. The high-rate cycling performance at a 1 C rate also shows a similar trend to the 0.1 C rate, with Co_3_O_4_@C still maintaining a high capacity of 989.3 mAh g^−1^ for 500 cycles.

**Figure 7:**
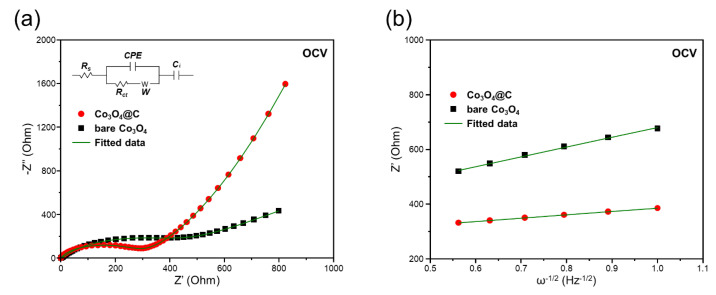
(**a**) Nyquist plots of bare Co_3_O_4_ and Co_3_O_4_@C at OCV state and (**b**) the corresponding relationships between Z′_real_ and *ω^−^*^1/2^ at a low-frequency region.

Figure 7a presents the Nyquist plots for the Co_3_O_4_@C and bare Co_3_O_4_ samples at open-circuit voltage (OCV). Each plot includes a semicircle, representing the charge transfer resistance (*R_ct_*) at the electrode/electrolyte interface, along with a sloped line indicative of Warburg resistance (W_o_), linked to lithium-ion diffusion within the solid electrode [47,48]. The *R_ct_* for bare Co_3_O_4_ was about 473 Ω, significantly higher than the 352 Ω observed for Co_3_O_4_@C. This reduction in R_ct_ highlights the enhanced conductivity and lower resistance of the Co_3_O_4_@C composite due to the carbon integration. The detailed supplementary parameters used for simulating the experimental EIS spectra, along with the equivalent circuit shown in Figure 7a, are available in Table S2.

*Z′*_*real*_ = *R*_*s*_ + *R*_*ct*_ + *σ*_*ω*_*ω*^−1/2^ (1)

(2) $$D_{\mathrm{Li}}=0.5\;{\left(\frac{\mathrm{RT}}{{\mathrm{An}}^{2}F^{2}\sigma_{\omega }C}\right)}^{2}$$

where R is the gas constant, T is the absolute temperature (in Kelvin), A is the electrode surface area, n is the number of electrons involved in the redox reaction, F is the Faraday constant, *σ_ω_* is the Warburg coefficient, and C is the concentration of lithium ions [49,50].

The lithium-ion diffusion coefficients (D_Li+_) for the bare Co_3_O_4_ sample and the Co_3_O_4_@C sample were determined by plotting Z′_real_ towards *ω^−^*^1/2^ in Figure 7b through Equation (1) to calculate the *σ_ω_*. After substituting the obtained *σ_ω_* values into Equation (2), the D_Li+_ was estimated to be 1.18 × 10^−14^ cm^2^ s^−1^ for bare Co_3_O_4_ and 1.01 × 10^−13^ cm^2^ s^−1^ for Co_3_O_4_@C at the OCV state. These findings suggest that Co_3_O_4_@C exhibits superior electrochemical kinetics and reduced resistance for lithium-ion redox reactions relative to bare Co_3_O_4_. The enhanced performance of Co_3_O_4_@C is likely due to the influence of carbon-compositing and the distinctive microsphere morphology. The carbon layer provides improved electrical conductivity, reduces charge transfer resistance, and offers additional active sites for lithium-ion storage, facilitating faster ion diffusion. Furthermore, the porous structure of the microspheres allows for better electrolyte penetration, enhancing the overall electrochemical performance.

## 4. Conclusions

In this study, carbon composites of Co_3_O_4_@C microspheres were successfully synthesized and demonstrated superior electrochemical performance compared to bare Co_3_O_4_. The presence of carbon can enhance conductivity. In addition, the unique porous structure of the Co_3_O_4_@C microspheres allowed for efficient electrolyte penetration and provided additional active sites for lithium-ion storage. This resulted in higher discharge capacities, improved ion diffusion, and better cycling stability of Co_3_O_4_@C microspheres. The Co_3_O_4_@C composite achieved a higher reversible discharge capacity and faster redox kinetics, making it a promising candidate for advanced LiB anodes. Overall, the composites of Co_3_O_4_@C microspheres exhibited superior electrochemical performance compared to bare Co_3_O_4_, making them a promising candidate for next-generation lithium-ion battery anodes and a promising model for developing other TMO-based anode materials.

## Data Availability

The original contributions presented in this study are included in the article as a part of the Supplementary Materials, and further inquiries can be addressed to the corresponding author.

## Supplementary Materials

The following supporting information can be downloaded at https://www.mdpi.com/article/10.3390/ma17235702/s1: Figure S1: XRD spectra from the intermediate products of the Co_3_O_4_@C*2 synthetic route; Table S1: Cycling performances of the present work and previously reported Co_3_O_4_-based electrodes for LIBs; Table S2: Fitting parameters for EIS spectra of bare Co_3_O_4_ and Co_3_O_4_@C in LIBs. Reference [51] is cited in the supplementary materials.
