# Supplementary material for: Enhanced Electrochemical Performance of Carbon-Composited Co3O4 Microspheres as Anode Materials for Lithium-Ion Batteries
Source: Materials (Basel). 2024 Nov 21;17(23):5702. doi: 10.3390/ma17235702 (PMC11642568; doi:10.3390/ma17235702)
Supplement: Supplementary file 1 [file materials-17-05702-s001.zip › materials-3293655-supplementary.pdf]

**Electronic supplementary information (ESI)**

Table S1. Cycling performances of the present work and previously reported $Co_3O_4$-based electrodes for LIBs.

| Electrode | Voltage range (V) | Current density (mA $g^{-1}$) | Reversible capacity (mA h $g^{-1}$) | Cycle number | Ref. |
|---|---|---|---|---|---|
| $Co_3O_4$-GO | 0.01-3.0 | 100 | 851.3 | 250th | [1] |
| H-Co3O4@IEH-Graphene | 0.01-3.0 | 100 | 840 | 250th | [2] |
| Hollow Co-$Co_3O_4$@CNTs | 0.01-3.0 | 200 | 806.7 | 200th | [3] |
| CNTs/$Co_3O_4$ p-NR | 0.01-3.0 | 500 | 1083 | 140th | [4] |
| $Co_3O_4$ nanobelts | 0.01-3.0 | 100 | 980 | 60th | [5] |
| $Co_3O_4$ PCCs | 0.01-3.0 | 89 | 1037.4 | 100th | [6] |
| $Co_3O_4$ hollow tetrahedra | 0.01-3.0 | 50 | 1196 | 60th | [7] |
| $Co_3O_4$ nanoparticles | 0.01-3.0 | 100 | 833.4 | 100th | [8] |
| $Co_3O_4$@PNGC | 0.01-3.0 | 89 | 1295.3 | 60th | [9] |
| $Co_3O_4$@C microspheres | 0.01-3.0 | 89<br>890 | 1557.4<br>989.3 | 200th<br>500th | This work |

Table S2. Fitting parameters for EIS spectra of bare $Co_3O_4$ and $Co_3O_4$@C in LIBs.

[The symbols are the resistance of the electrolyte ($R_s$), film resistance of SEI layer ($R_f$), charge transfer resistance ($R_{ct}$) and capacitance of SEI layer (CPE1), double layer capacitance (CPE2) at the interface between electrode and electrolyte and Warburg resistance ($W_o$)]

| | OCV State | |
|---|---|---|
| | bare $Co_3O_4$ | $Co_3O_4$@PNGC |
| $R_s$ (Ω) | 2.22 | 2.22 |
| CPE1 (F) | 0.86 | 0.98 |
| $R_f$ (Ω) | 14359 | 14053 |
| CPE2 (F) | 0.83 | 0.91 |
| $R_{ct}$ (Ω) | 473 | 302.8 |
| $W_o$ (Ω) | 0.0045 | 0.0013 |

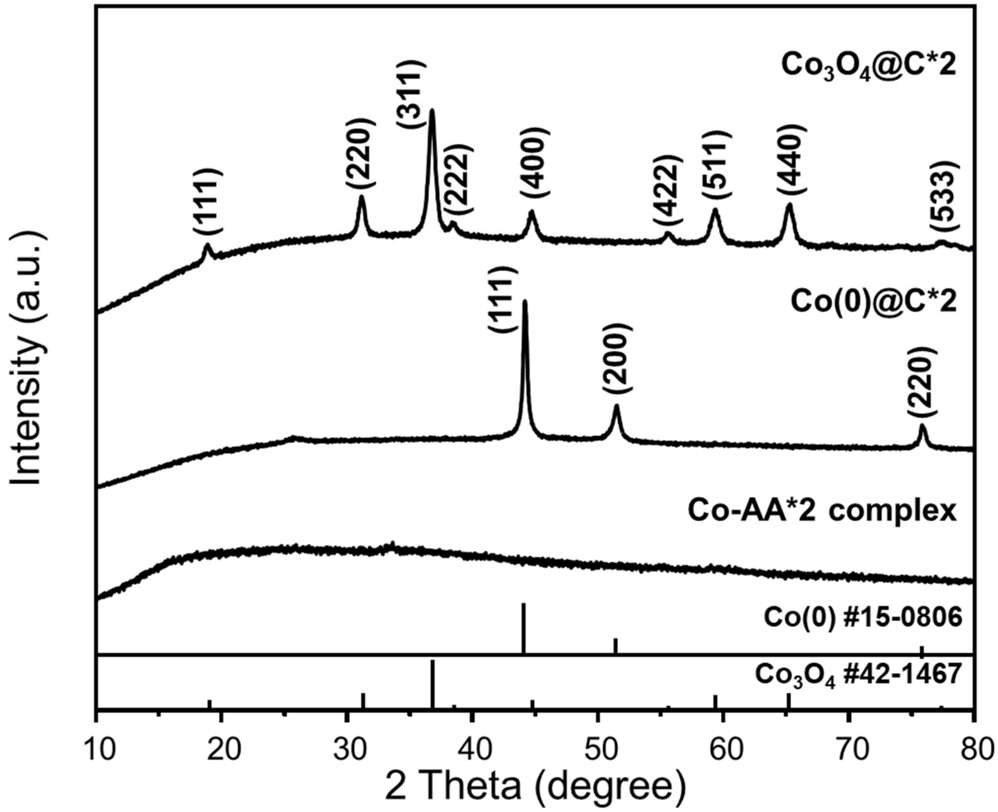


Figure S1. XRD spectra from the intermediate products of the $Co_3O_4$@C*2 synthetic route

## References


1. Wang, W.; Cui, H.; Cui, J.; Wang, T.; Sha, J.; Liu, G., $Co_3O_4$ anode for lithium-ion batteries coupled by polyoxometalates and graphene for boosting ion-/electron-conductivity. *Journal of Alloys and Compounds* **2023,** 967, 171817.
2. Wu, D.; Wang, C.; Wu, H.; Wang, S.; Wang, F.; Chen, Z.; Zhao, T.; Zhang, Z.; Zhang, L. Y.; Li, C. M., Synthesis of hollow Co3O4 nanocrystals in situ anchored on holey graphene for high rate lithium-ion batteries. *Carbon* **2020,** 163, 137-144.
3. Li, Y.; Fu, Y.; Liu, W.; Song, Y.; Wang, L., Hollow Co-$Co_3O_4$@CNTs derived from ZIF-67 for lithium ion batteries. *Journal of Alloys and Compounds* **2019,** 784, 439-446.
4. Li, X.; Tian, X.; Yang, T.; Song, Y.; Liu, Z., Hierarchically multiporous carbon nanotube/$Co_3O_4$ composite as an anode material for high-performance lithium-ion batteries. *Chemistry–A European Journal* **2018,** 24, (54), 14477-14483.
5. Huang, H.; Zhu, W.; Tao, X.; Xia, Y.; Yu, Z.; Fang, J.; Gan, Y.; Zhang, W., Nanocrystal-constructed mesoporous single-crystalline $Co_3O_4$ nanobelts with superior rate capability for advanced lithium-ion batteries. *ACS applied materials & interfaces* **2012,** 4, (11), 5974-5980.
6. Chen, F.; Yuan, Y.; Ye, L.; Zhu, M.; Cai, G.; Yin, S.; Yang, J.; Guo, S., $Co_3O_4$ nanocrystalline-assembled mesoporous hollow polyhedron nanocage-in-nanocage as improved performance anode for lithium-ion batteries. *Materials Letters* **2019,** 237, 213-215.

7. Tian, D.; Zhou, X.-L.; Zhang, Y.-H.; Zhou, Z.; Bu, X.-H., MOF-derived porous $Co_3O_4$ hollow tetrahedra with excellent performance as anode materials for lithium-ion batteries. *Inorganic chemistry* **2015,** 54, (17), 8159-8161.
8. Wan, H.; Liu, Y.; Zhang, H.; Zhang, W.; Jiang, N.; Wang, Z.; Luo, S.; Arandiyan, H.; Liu, H.; Sun, H., Improved lithium storage properties of $Co_3O_4$ nanoparticles via laser irradiation treatment. *Electrochimica Acta* **2018,** 281, 31-38.
9. Wang, G.; Zhang, M.; Deng, Z.; Zhang, X.; Huo, L.; Gao, S., Poplar branch bio-template synthesis of mesoporous hollow $Co_3O_4$ hierarchical architecture as an anode for long-life lithium ion batteries. *Ceram. Int.* **2020,** 46, 29033-29040.
